# Insights into Retinal Metastasis from Systemic Carcinoma: A Systematic Review of Clinical and Multimodal Imaging Characteristics

**DOI:** 10.3390/jcm13206037

**Published:** 2024-10-10

**Authors:** Ling-Uei Wang, Tzu-Han Hsieh, Fang-Ting Chen, Yun-Ju Chen, Jia-Kang Wang, Yung-Ray Hsu

**Affiliations:** 1 Department of Ophthalmology, Far Eastern Memorial Hospital, New Taipei City 220216, Taiwan; 2 Department of Ophthalmology, National Taiwan University Hospital, Taipei 100226, Taiwan; 3 College of Medicine, National Taiwan University, Taipei 100233, Taiwan; 4 Department of Electrical Engineering, Yuan Ze University, Taoyuan 320315, Taiwan

**Keywords:** retina, neoplasm metastasis, carcinoma, neoplasms

## Abstract

**Background/Objectives**: Retinal metastasis (RM) is an exceptionally rare clinical condition, comprising less than 1% of ocular metastasis cases. This study aims to investigate the clinical features of RM originating from systemic carcinoma. **Methods**: A systematic review (PROSPERO ID: CRD42022344724). **Results**: The systematic review revealed 58 cases involving 61 eyes. Most of the cases (82.8%) had a known primary malignancy before RM was diagnosed. The main primary sites were the lung (45.8%), gastrointestinal tract (23.7%), and breast (16.9%). The lung was the most common (80.0%) carcinoma in cases with unknown primary sites. The median survival after RM diagnosis was 11 months. The main clinical patterns were patchy retinal infiltrates (35.7%), an elevated retinal mass (63.8%), and punctate retinal infiltrates (7.1%). Subretinal fluid, vitreous seeding, and choroidal invasion were noted in 57.4%, 41.0%, and 6.6% of eyes, respectively. Key multimodal imaging features were early hypofluorescence with mid-to-late hyperfluorescence on fluorescein angiography (84.6%) and hyper-reflectiveness on optical coherence tomography (70.8%). A final best-corrected visual acuity of ≤20/200 was noted in 66.7% of the eyes. **Conclusions**: Patchy retinal infiltrates, an elevated retinal mass, and punctate retinal infiltrates are the major clinical patterns of RM from systemic carcinoma. The primary carcinoma sites are the lung, gastrointestinal tract, and breast. Systemic and visual prognoses were unfavorable.

## 1. Introduction

Most cases of ocular metastasis from systemic cancer appear in the uveal tracts (>98%), especially the choroid. On the contrary, retinal metastasis (RM) is extremely rare, accounting for <1% of cases of ocular metastasis [1]. This rarity may be attributed to the difference in vascularization between the choroid and retina. The retina is supplied by a single central retinal artery (CRA) that provides blood to the inner layers and is distributed throughout the retina, whereas the highly vascularized choroid is supplied by multiple choroidal arteries.

RM is an umbrella term referring to the invasion of the retina by remote systemic malignant cells. Among various types of cancer, carcinoma is the most typical entity of solid organ tumors. Meanwhile, carcinomatous RM is also more commonly seen than RM from melanoma, lymphoma, or leukemia cells [2,3,4,5,6,7]. Given the rarity of RM, the literature on RM includes isolated case reports and a few case series. Those studies have enrolled patients with heterogeneous clinical entities and have applied inconsistent inclusion and exclusion criteria [2,3,8]. Since the clinical outcomes and biological behaviors associated with systemic malignancies of different cell types vary widely, analyzing a single type of malignancy is crucial to obtaining a precise and in-depth understanding of RM.

This systematic review investigates RM from systemic carcinoma and describes this disease entity’s clinical features and outcomes.

## 2. Materials and Methods

In accordance with preferred reporting items for systematic reviews and meta-analyses (PRISMA) recommendations [9], we selected articles from PubMed, CINAHL, and Google Scholar that were published on or before May 31, 2024. This systematic review has been prospectively registered in PROSPERO (registration ID: CRD42022344724), entitled “Systemic and Ocular Features of Retinal Metastatic Carcinoma”. We used 4 search terms to identify eligible articles: “retinal metastasis”, “retina metastasis”, “metastasis to the retina”, and “metastatic retinal mass”. Three reviewers (LUW, THH, and YRH) reviewed the titles and abstracts independently. After removing duplicate articles, the full texts of the remaining articles were obtained and thoroughly reviewed. Disagreement between the reviewers was resolved through the re-evaluation of the manuscript and further discussion. Prespecified inclusion and exclusion criteria are shown in Table 1 and applied to ensure study quality. Only studies that provided sufficient relevant information on patients’ demographic profiles, main ocular features, adjunctive ocular features, and systemic features of RM were enrolled. Only peer-reviewed English language publications were reviewed. All the collected data were compiled into an Excel spreadsheet (Excel for Mac version 16.48, Microsoft, Washington, DC, USA), and descriptive statistics were conducted on the spreadsheet. A Kaplan–Meier survival analysis was performed using jamovi (the jamovi Project. n.d. jamovi (Version 2.3.26) [Computer Software]. https://www.jamovi.org (accessed on 30 June 2024)).

## 3. Results

### 3.1. Systemic Features

**Figure 1:**
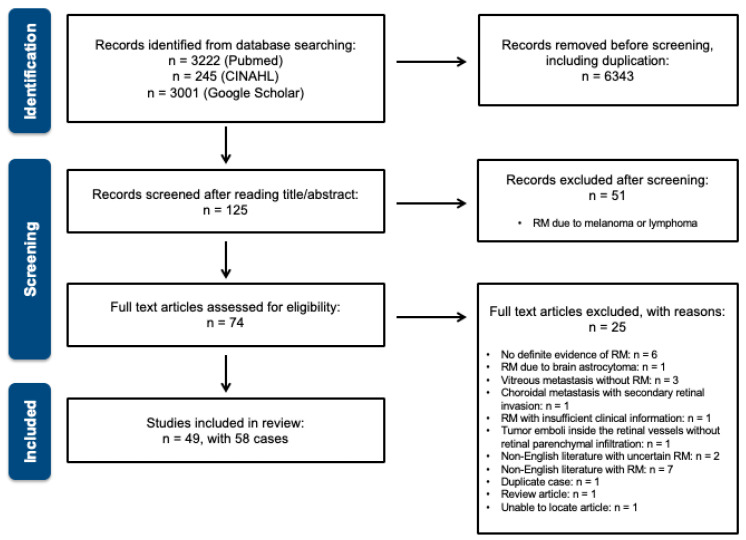
PRISMA flow diagram.

**Figure 2:**
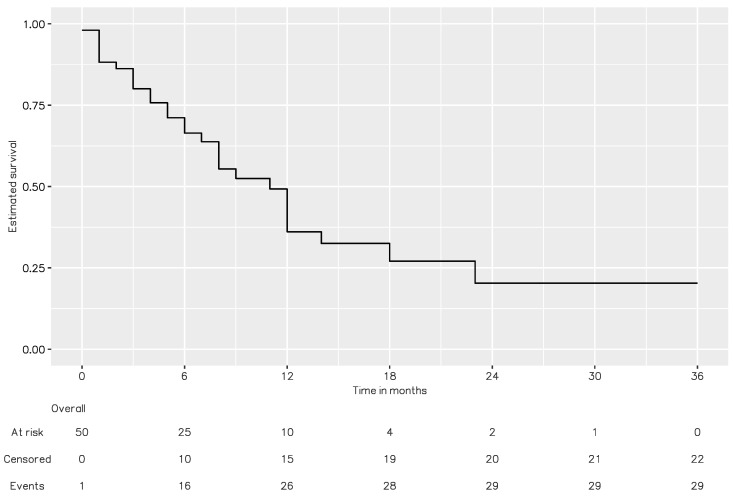
Kaplan–Meier survival analysis of RM from systematic review. The median interval from RM diagnosis to death was 11 months.

Overall, 49 studies involving 58 cases were eligible for review and the PRISMA flow diagram is shown as Figure 1 (the complete literature list is provided in the Supplementary Table S1). The patients’ systemic profiles are described in Table 2. The studies included 36 men (62.1%) and 22 women (37.9%) with a median age of 59 years (IQR = 13.5; range = 15–80). Most (*n* = 55, 94.8%) presented with unilateral RM. The three most common primary carcinoma sites were the lung (*n* = 27, 45.8%), GI tract (*n* = 14, 23.7%), and breast (*n* = 10, 16.9%). The patient’s primary diagnosis, metastasis, and mortality profiles are summarized in Table 3. Primary carcinoma diagnosis preceded ocular manifestations in 82.8% of the cases (*n* = 48), with varied intervals (median = 18.0 months; IQR = 27.0; range = 1–214). Among the cases in which ocular manifestations preceded the primary diagnosis (*n* = 10, 17.2%), lung cancer (*n* = 8, 80.0%) was the most common primary diagnosis. Systemic metastasis was noted in 45 (86.3%) patients. Twenty-two patients (59.4%) had an involvement of more than two solid organs, excluding lymph node metastasis. The lymph nodes, lungs, and brain were the three main sites involved (51.2%, 46.3%, and 46.3%, respectively). Considering the treatment after RM diagnosed, 82.4% received chemotherapy among 17 patients with systemic treatment according to the primary carcinoma and 71.4% received radiotherapy for 28 patients receiving local treatment. Overall, 56.8% of the patients died during the follow-up. A Kaplan–Meier survival analysis (Figure 2) revealed that the median interval from RM diagnosis to death was 11 months.

### 3.2. Ocular Features

**Figure 3:**
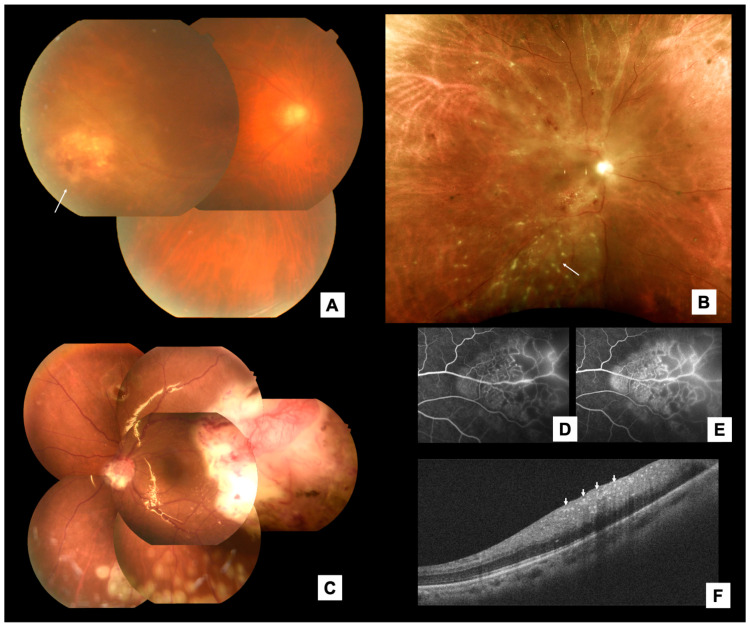
Representative of 3 retinal patterns and multimodal image findings of RM. Adapted from Chen [12]. [Figure 1]. (**A**) Patchy retinal infiltrate. (**B**) Punctate retinal infiltrate. (**C**) An elevated retinal mass with tortuous retinal neovascularization. (**D**,**E**) Intrinsic vascularity on fluorescein angiography at mid-phase (2:49) and late phase (9:18). (**F**) Inner retina hyper-reflective changes (arrows) on OCT.

The patients’ ocular features are summarized in Table 4. We divided the clinical appearance of RM into three main clinical patterns, as in Figure 3: [12] patchy retinal infiltrates (*n* = 20, 35.7%); an elevated retinal mass (*n* = 20, 63.8%); and punctate retinal infiltrates (*n* = 4, 7.1%). Subretinal fluid (SRF) was noted in 35 (57.4%) eyes. Serous RD (SRD), rhegmatogenous retinal detachment (RRD), and tractional RD (TRD) were noted in 47.5%, 8.2%, and 1.6% of eyes, respectively. Vitreous seeds were noted in 25 (41.0%) eyes. Choroidal invasion was noted in four (6.6%) eyes. A pathological diagnosis was obtained in 43 (70.5%) eyes. Pars plana vitrectomy (PPV) with a vitreous/retinal/SRF biopsy was performed on 23 (53.5%) eyes. A fine-needle aspiration biopsy was performed on seven (16.3%) eyes. An initial misdiagnosis was noted in 21 cases (37.5%), of which 13 cases (61.9%) were recorded as infectious retinitis. The mean best corrected visual acuity (BCVA) logarithm of the minimum angle of resolution (logMAR) at presentation and the end of follow-up was 1.06 (Snellen equivalent: 20/230) and 1.73 (Snellen equivalent: 20/1074), respectively. A Snellen BCVA of ≤20/200 was noted in 18 (37.5%) and 22 (66.7%) eyes at the initial and final visits, respectively.

The multimodal imaging findings were investigated. The most common FA pattern was early hypofluorescence with mid/late-phase hyperfluorescence (lesion staining/leakage) (*n* = 22, 84.6%). Intrinsic intralesional vascularity was noted in 10 (38.5%) eyes. The lesions were predominantly hyper-reflective (*n* = 17, 70.8%) on OCT, hyperechoic (*n* = 9, 100%) on a B-scan ultrasonography, and hypo-autofluorescent (*n* = 5, 100%).

## 4. Discussion

Cases of RM were first reported before 1960 [13,14,15]; since then, oncologic therapies, multimodal ophthalmic imaging, and surgical procedures have advanced considerably. However, the management of RM still needs to be improved. As RM is rare, relevant information on ocular presentations, protocols of vitreoretinal biopsies, primary tumor and systemic metastasis profiles, and prognosis are scarce. Although most of the publications included in our review were case reports, Shields et al. presented four cases of histologically diagnosed RM from systemic carcinoma among a series of eight cases of RM [2]. More recently, Gascon et al. presented a series of one biopsy-confirmed and two clinically presumed cases of carcinomatous RM [3]. The current study presents a rigorous systematic review with predefined criteria.

This systematic review revealed three key systemic features of RM. First, similar to uveal metastasis, lung and breast carcinoma are the most common types of RM among male and female patients, respectively. Meanwhile, cases of RM originating in the GI tract are among the three leading diagnoses in both male and female patients. Therefore, we suggest that the examinations of chest/abdominal CT, mammography/breast echography, and panendoscopy should be prioritized in cases without cancer history. Second, the intervals from primary cancer diagnosis to RM varied widely: 38.4% of the patients presented with RM within 1 year of their primary diagnosis, and 35.9% presented with RM more than 2 years after their primary diagnosis. Therefore, a long or relatively stable preceding malignancy does not preclude the possibility of RM. In nearly 20% of the cases, ocular manifestations preceded the primary diagnosis; among them, lung cancer was the most common primary diagnosis (80%), reflecting its aggressive nature. Third, the systemic prognoses of patients with RM are often poor. Systemic metastasis was common (86.3%) and was often multiple (59.4%). The Kaplan–Meier analysis revealed that the median interval from RM diagnosis to death was 11 months. This information is crucial to decisions related to holistic care systemic management and communication among physicians, patients, and family members.

Our systematic review also revealed several key ocular characteristics of RM. Three distinct clinical patterns were identified. Although the diagnosis of RM is relatively straightforward when RM manifests as an elevated retinal mass, the other two patterns can masquerade as uveitis entities [16]. Patchy retinal infiltrates may be accompanied by a retinal hemorrhage and exudates, often mistaken for infectious retinitis [11,17,18,19]. When such lesions appear in patients with cancer who are immunocompromised, cytomegaloviral or toxoplasmic retinitis is often suspected. Punctate retinal infiltrates, the least common of the three patterns, can be mistaken as noninfectious uveitis, such as those that appear in sarcoidosis or Behcet disease. Physicians should be aware of the possibility of RM when initial investigations or treatment responses are suboptimal.

The biological nature of RM can be assessed using the clinical information and multimodal imaging findings collected in this study. Most retinal lesions occurred in the posterior pole or midperiphery, with predominant inner-retinal hyper-reflective infiltration shown on OCT, reflecting tumor invasion through the CRA. Since the CRA is a terminal branch of the systemic circulation, RM presumably occurs in the case of a large metastatic tumor burden, the entry of malignant cells into the CRA, and the partial disruption of the inner blood–retinal barrier (BRB), which explains the rarity, multiple metastases, and poor systemic prognosis of RM. Interestingly, choroidal involvement was uncommon (6.6%). As the hypoautofluorescence profile indicates, outer retinopathy or retinal pigment epithelium (RPE) disturbance is uncommon. The intact tight junction of the RPE may protect against cancer cell breakthrough. Meanwhile, tumors with selected adhesion molecules may be more adapted to cross the BRB and invade the retinal tissues. Finally, some cases with concurrent chorioretinal metastasis were possibly ignored when the choroidal component was more impressive and easily diagnosed.

The hypermetabolic state of these carcinoma cells can be demonstrated by the late fluorescent staining/leakage (84.6%) with intrinsic vascularity (38.5%). This may explain the aggressive intraocular behaviors of RM. Although SRD is more common, RRD [3,20] and TRD [17] are also possible. First, as reported by Taubenslag et al. [11], retinal breaks can form due to the contraction of the retinal tumor. Second, densely infiltrated vitreous skirt contraction from tumor infiltration may lead to retinal breaks. Third, prior tumor tumefaction may cause breaks at the tumor’s border. These three mechanisms may contribute to the relatively large number (8.2%) of RRD in RM compared to < 1% RRD in choroidal metastases [21]. Following RM, tumor invasion to the iris with neovascular glaucoma is not uncommon. As reported by Saornil et al. [22], Spraul et al. [10], and Koenig et al. [23], the diagnosis was established by examining the enucleated painful blind eye. This reflects the poor visual prognoses of eyes with RM.

Whether pathological evidence is required for RM diagnosis remains unclear. Physicians should evaluate the risks and benefits of surgical procedures on a case-by-case basis. Systemic workup and collaboration with the oncologist are also crucial. In most cases, however, histological evidence from ocular sampling provides important information to guide further management. Indirect ophthalmoscopy and operating microscope-guided fine-needle aspiration biopsies with 25-gauge needles, as described by Shields et al., are well-established procedures with low complication rates [24]. However, because of advances in the vitrectomy systems, PPV remains the most common procedure for obtaining the specimen. It is beneficial for cases with dense vitreous infiltration with a poor fundal view and with vitreoretinal complications such as RRD or TRD and for small retinal lesion biopsies, which require precise intraoperative localization. In cases of significant vitreous infiltration, a vitreous biopsy suffices. Sometimes, a retinal biopsy with the following surgical techniques may be necessary. A limited retinectomy with a tumor-free margin and en bloc retinal tumor excision can be performed in far-peripheral giant retinal tear RRD due to cancer cell infiltration or the highly elevated retinal mass with bullous SRD [25]. In most posterior lesions, however, tumor excision may result in permanent structural complications [26]. Therefore, a conservative intralesional incisional biopsy is often more appropriate [18,27]. In some cases, by involving vitreoretinal surface membranes or cell aggregates, membranes/aggregates can be lifted using forceps and gently aspirated using a backflush needle. Finally, a chandelier endoillumination-assisted vitrectomy with the creation of focal RD with a 41-gauge cannula followed by local retinectomy [28] is another possible option.

The management of RM remains challenging. Although systemic chemotherapy is usually used to treat multiple organ metastases, it may be ineffective for intraocular lesions. For patients with advanced cancer, maintaining spiritual well-being and quality of life is crucial. Moreover, multidisciplinary management is necessary to reach a proper treatment [29]. If a patient’s remaining visual acuity can be salvaged and a painful blind eye can be prevented, the patient’s fear and anxiety can be minimized. Therefore, ocular therapy should be administered using simple, noninvasive, and short procedures. Although an intravitreal injection (IVI) of anti-vascular endothelial growth factor (VEGF) is a short and simple procedure [3], it is invasive, and its efficacy depends on the primary tumor type. If IVI of anti-VEGF fails, injecting other chemotherapy agents into the eye is often not feasible because of insufficient information regarding the dosage and retinal toxicity of these medications. Photodynamic therapy is another feasible option [3,30]. As most RM lesions are vascularized according to FA profiles, the effect of the tumor size control and reduction of exudation can be anticipated. Since an ophthalmologist administers photodynamic therapy, a detailed explanation and follow-up are preferable. Finally, the external beam radiation therapy is widely used to control intraocular tumors [28,31,32,33,34]. It is an advantageous, non-invasive treatment option for concurrent brain metastasis. However, patients who undergo such therapy should be monitored for secondary radiation retinopathy. Overall, individually tailored treatment plans are crucial.

This study has some limitations. First, with the prespecified criteria for systematic reviews, some publications were inevitably excluded because of insufficient clinical data. Due to the generally poor systemic condition of patients with RM, missing data for various clinical parameters and multimodal imaging were common. Meanwhile, misdiagnosis was possible in some reports without pathological evidence. Second, because this study focused only on RM from systemic carcinoma, other major sources of RM, such as melanoma or lymphoma, were excluded. Therefore, comparing carcinomatous RM with other different types of RM was not possible. Nevertheless, this study has several strengths of note. The information presented in this study provides clear insights into the various clinicopathological findings of RM. In addition, by focusing only on metastatic carcinoma, we avoided the confusion surrounding the inconsistent definitions of RM employed in previous studies and limited our analysis to a uniform disease entity. The data were extracted from three major databases using stringent prespecified criteria. A complete presentation of clinical features, prognosis, biopsy methods, and treatment options were summarized herein.

## 5. Conclusions

RM is a rare but severe disease. The main primary carcinoma sites were the lung, GI tract, and breast, and the three main ocular manifestations were patchy retinal infiltrates, an elevated retinal mass, and punctate retinal infiltrates. These manifestations may present as uveitis masquerade syndromes. Although pathological diagnosis is preferable, combining systemic information and multimodal imaging may support the clinical diagnosis. The treatment of RM should be individually tailored and focus on holistic care and quality of life.

## Figures and Tables

**Table 1:** Inclusion and exclusion criteria.

**Inclusion Criteria**	
Study design: full articles, case reports/series, letters to the editor.	
Sufficient relevant information: the articles must provide sufficient information for all the following categories.	
1. Demographic profiles	Manuscript should contain complete information regarding the following: a. Age. b. Sex. c. Laterality.
2. Diagnostic features	Manuscript should contain complete information regarding the following: a. Patterns of retinal lesions. b. Sizes of retinal lesions (<1-, 1–3-, or >3-disc diameter). c. Distribution of retinal lesions (macula, juxtapapillary region, mid-periphery, far periphery). d. Diagnosis (pathological or clinical).
3. Adjunctive ocular features	Manuscript should contain information related to at least 2 of the following: a. Presence of subretinal fluid (serous RD, rhegmatogenous RD, tractional RD). b. Presence of vitreous seeds. c. Choroidal involvement. d. Visual acuity (presenting and final). e. Multimodal imaging findings (OCT, B-scan ultrasonography, FA).
4. Systemic features	Manuscript should contain information related to at least 2 of the following: a. Primary carcinoma site. b. Primary carcinoma status before ocular presentation (known or unknown). c. Systemic metastasis involving nonocular organs. d. Survival/death and follow-up time.
**Exclusion criteria**	
Study design: review articles without new cases; non-English-language literature.	
Manuscript information:	
1. Retinal metastasis from systemic malignancy that is not carcinoma (e.g., lymphoma, melanoma, leukemia, or sarcoma). 2. Reports of elusive description of the diagnosis, such as “intraocular metastasis” or “ocular metastasis” without definite evidence of retinal metastasis. 3. Reports of choroidal, vitreous, or optic-nerve metastasis with secondary retinal complications, such as retinal detachment, retinal vasculitis, or macular edema, without retinal infiltration of malignant cells. 4. Clinical evidence suggesting choroidal metastasis with secondary retinal invasion. 5. Tumor emboli inside the retinal vessels without retinal parenchymal infiltration. 6. Manuscripts that failed to provide sufficient information per the inclusion criteria. 7. Non-human studies. 8. Duplicate cases identified according to identical clinical history and imaging findings.	

**Table 2:** Basic demographic data and primary carcinoma sites of patients with retinal metastatic carcinoma (RM).

	**N**	**%**
Demographic Profile	58	100
Sex		
Male	36	62.1%
Female	22	37.9%
Age	Median = 59; IQR = 52–65.5; Range = 15–80	
Laterality		
Unilateral	55	94.8
Bilateral	3	5.2
Primary carcinoma site	59	100
Lung	27	45.8%
Small-cell lung carcinoma	8	13.6%
Adenocarcinoma	7	11.9%
Squamous cell carcinoma	6	10.2%
Others	6	10.2%
Gastrointestinal tract	14	23.7%
Colorectum *	8	13.6%
Esophagus	3	5.1%
Stomach	3	5.1%
Breast *	10	16.9%
Others	8	13.6%
Nasopharynx	1	1.7%
Larynx	1	1.7%
Bile duct	1	1.7%
Kidney	1	1.7%
Urinary bladder	1	1.7%
Prostate	1	1.7%
Uterine	1	1.7%
Undetermined	1	1.7%
Leading diagnoses according to sex		
Male	36	100
Lung	20	55.6
Colorectum	6	16.7
Stomach	2	5.6
Female	22	100
Breast	12	54.5
Lung	5	22.7
Colorectum	2	9.1

* Double primary tumors were noted in one case (concurrent breast and colon adenocarcinoma with Muir–Torre syndrome, Spraul 1995 [10]).

**Table 3:** Primary diagnosis, metastasis, and mortality profiles of patients with RM from systemic carcinoma.

	N	%
Primary carcinoma diagnosis before RM		
Yes	48	82.8
Interval from primary cancer to RM (months, n = 39) *	Median = 18.0; IQR = 7.5–34.5; Range = 1–214	
<6 M	8	20.5
6 M–12 M	7	17.9
13 M–24 M	10	25.6
25 M–36 M	6	15.4
>36 M	8	20.5
No	10	17.2
Lung cancer	8	80.0
Esophageal cancer	1	10.0
Undetermined ^†^	1	10.0
Systemic metastasis ^#^	52	100.0
Yes ^$^	45	86.3
Lymph node or solid organs (n = 41)		
Lymph node	21	51.2
Brain	19	46.3
Lung	19	46.3
Liver	16	39.0
Bone	14	34.1
Adrenal gland	7	17.1
Others ^	12	29.3
Number of solid organs involved (n = 37) **		
1	15	40.5
2	8	21.6
≥3	14	37.8
No	7	13.7
Systemic treatment after RM diagnosed	23	100.0
Yes	17	73.9
Chemotherapy	14	82.4
Immunotherapy	3	17.6
Targeted therapy	2	11.8
No	6	26.1
Ocular treatment after RM diagnosed	35	100.0
Yes	28	80.0
Radiotherapy	20	71.4
Intravitreal injection of anti-VEGF	4	14.3
Enucleation	3	10.7
Brachytherapy	2	7.1
Intravitreal injection of chemotherapy	1	3.6
Photodynamic therapy	1	3.6
No	7	20.0
Mortality during follow-up ^‡^	51	100.0
Yes	29	56.8
Time from RM to death (months), Kaplan–Meier survival analysis	Median = 11.0; IQR = 8–18	

* In 48 cases where systemic diagnosis preceded ocular manifestations, interval data were only available for 39. ^†^ Undetermined case: pathology favored non-small-cell lung carcinoma, but no primary tumor was detected, Taubenslag 2015. [11] ^#^ Among the 58 cases, documented systemic metastasis profiles were available for 52; data not mentioned in the manuscript: *n* = 3; unknown, autopsy declined by family: *n* = 2; double primary cancer, not considered metastasis: *n* = 1. ^$^ Out of the 45 cases involving systemic metastasis, information on specific organ involvement was described in 41 cases. As some patients had multiple systemic metastases, total percentages may exceed 100%. ^ This includes spleen, *n* = 2; pancreas, *n* = 2; kidney, *n* = 1; peritoneum, *n* = 2; skin, *n* = 2; thyroid, *n* = 1; heart, *n* = 1; mediastinum, *n* = 1; ** Cases involving isolated lymph node invasion (*n* = 4) were not included; therefore, the total *n* = 37. ^‡^ Only 51 cases had documented survival information; some cases (*n* = 4) did not document relevant information in the manuscript; 2 patients were too sick to keep follow-up.

**Table 4:** Ocular features of RM from systemic carcinoma (61 eyes).

	**N**	**%**
Retinal pattern *	56	100
Patchy retinal infiltrate	20	35.7
Elevated retinal mass	32	57.1
Punctate retinal infiltrate	4	7.1
Lesion size *	57	100
Lesion ≤ 1 disc diameter (DD)	16	28.1
1DD < lesion ≤ 3DD	21	36.8
Lesion > 3DD	20	35.1
Location **	57	100
Macula or juxtapapilla	36	63.2
Mid-periphery	26	45.6
Far periphery	9	15.8
Adjunctive features	61	100
Subretinal fluid	35	57.4
Serous RD	29	47.5
Rhegmatogenous RD	5	8.2
Tractional RD	1	1.6
Vitreous seeding	25	41.0
Choroidal invasion	4	6.6
Diagnosis of RM	61	100
Pathology proven	43	70.5
Vitrectomy	23	53.5
with vitreous biopsy	14	32.6
with retinal biopsy	9	20.9
with subretinal fluid biopsy	1	2.3
Fine-needle aspiration biopsy	7	16.3
with vitreous biopsy	3	7.0
with retinal biopsy	4	9.3
Iris excisional biopsy	1	2.3
Enucleation	8	18.6
Autopsy	6	14.0
Clinically presumed	18	29.5
BCVA (Snellen) ^#^		
Presenting (n = 48)	Mean LogMAR = 1.06; equivalent to Snellen 20/230	
BCVA > 20/40	10	20.8
20/200 < BCVA ≤ 20/40	20	41.7
BCVA ≤ 20/200	18	37.5
Final (n = 33)	Mean LogMAR = 1.73; equivalent to Snellen 20/1074	
BCVA > 20/40	5	15.2
20/200 < BCVA ≤ 20/40	6	18.2
BCVA ≤ 20/200	22	66.7
Multimodal imaging		
FA	26	100
Early hypoF with late hyperF	22	84.6
Intrinsic vascularity	10	38.5
OCT	24	100
Hyper-reflective lesion	17	70.8
Isoreflective lesion	7	29.2
B-scan ultrasonography	9	100
Hyperechoic lesion	9	100
Fundus autofluorescence	5	100
Hypo-autofluorescence	5	100

* Retinal pattern or lesion size information was unavailable for 5 and 4 eyes, respectively. ** Some lesions were multifocal; therefore, the total percentage may exceed 100%. Data were unavailable for 4 eyes, including 1 patient who did not receive an ocular exam and 3 patients whose information was not documented in the manuscript. ^#^ Presenting BCVA data were unavailable for 13 eyes (that did not receive ocular exams or of which the exam results were not documented in the manuscript); final BCVA data were unavailable for 28 eyes.

## Data Availability

Data are unavailable due to privacy or ethical restrictions.

## Supplementary Materials

The following supporting information can be downloaded at: https://www.mdpi.com/article/10.3390/jcm13206037/s1, Table S1: Complete List of the Literature in Systematic Review.
