# Supplementary material for: Insights into Retinal Metastasis from Systemic Carcinoma: A Systematic Review of Clinical and Multimodal Imaging Characteristics
Source: J Clin Med. 2024 Oct 10;13(20):6037. doi: 10.3390/jcm13206037 (PMC11508253; doi:10.3390/jcm13206037)
Supplement: Supplementary file 1 [file jcm-13-06037-s001.zip › jcm-3225935-supplementary.pdf]

Supplemental Table S1. Complete List of Literature in Systematic Review

Author/year	Sex/Age/Eye	Primary carcinoma/ Diagnosis before RM/ Interval of RM from primary, mo	Systemic metastases	Retinal pattern/ Presenting, final BCVA Subretinal fluid/Vitreous seeds	Diagnostic method	Systemic/Ocular treatment after RM diagnosed	Death/ Time, mo
Chen(1) 2024	M/52/R	Lung adenocarcinoma Unknown 0	Brain, liver, bone, lymph node	Patchy retinal infiltrate 20/40, LP No/ Yes	PPV with vitreous biopsy	Chemotherapy N/A	Yes 9
Chen(1) 2024	M/74/L	Lung squamous cell carcinoma Known 7	Brain, lung, lymph node	Patchy retinal infiltrate LP, HM Yes, RRD/ No	PPV with retinal biopsy	N/A	Yes 3.5
Chen(1) 2024	F/48/R	Breast cancer Known 132	Brain, lung, bone	Punctate retinal infiltrate CF, CF No/ Yes	PPV with vitreous biopsy	N/A	Yes 7
Chen(1) 2024	M/56/L	Lung squamous cell carcinoma Known 15	Brain, lymph node	Elevated retinal mass CF, CF Yes, RRD/ Yes	PPV with vitreous biopsy	N/A	No 15
Chen(1) 2024	M/60/L	Lung small cell carcinoma Unknown 0	Brain, liver, bone, lymph node, adrenal gland, spleen, pancreas	Patchy retinal infiltrate 20/32, 20/125 No/ No	Iris excisional biopsy (fellow eye)	N/A	Yes 8
Fairbanks(2) 2024	F/53/R	Papillary renal cell carcinoma Known 18	Lymph node	Patchy retinal infiltrate 20/60, N/M No/ Yes	PPV with vitreous and retinal biopsy	Immunotherapy Radiotherapy	Yes 18
Rahman(3) 2023	M/66/R	Lung squamous cell carcinoma Known 12	Bone	Patchy retinal infiltrate 20/60, NLP No/ Yes	PPV with vitreous biopsy	Immunotherapy Radiotherapy/Enucleation	No 26
Alabi(4) 2024	F/62/R	Lung small cell carcinoma Known 15	Mediastinum, bone	Elevated retinal mass LP, NLP Yes, SRD/ No	PPV with retinal biopsy and enucleation	N/A Enucleation	No 36
Ustaoglu(5) 2023	F/48/R	Lung small cell carcinoma Known 28	brain, lung, lymph node	Patchy retinal infiltrate 20/30, NLP Yes, SRD/ No	PPV with vitreous biopsy and enucleation	N/A	N/A

Marks(6)<br>2022	F/66/R	Small cell lung carcinoma<br>Known<br>8	No	Patchy retinal infiltrate<br>HM, N/M<br>Yes, SRD / No	PPV with subretinal fluid biopsy	Chemotherapy<br>Radiotherapy	No<br>14
Gascon(7)<br>2022	M/59/R	Colon adenocarcinoma<br>Known<br>33	Lung, liver	Elevated retinal mass<br>CF, HM<br>Yes, SRD / Yes	Clinical	N/A<br>Intravitreal injection of anti-VEGF	Yes<br>12
Gascon(7)<br>2022	M/63/R	Lung squamous cell carcinoma<br>Known<br>16	No	Elevated retinal mass<br>20/40, 20/32<br>Yes, RRD / Yes	PPV with vitreous biopsy	N/A<br>Radiotherapy	Yes<br>12
Gascon(7)<br>2022	M/51/R	Colon adenocarcinoma<br>Known<br>21	Liver, adrenal gland	Elevated retinal mass<br>CF, CF<br>No / No	Clinical	N/A<br>Intravitreal injection of anti-VEGF	Yes<br>1
Storey(8)<br>2021	M/73/L	Non-Small cell lung carcinoma<br>Known<br>24	No	Elevated retinal mass<br>20/60, 20/50<br>Yes, SRD / No	Clinical	N/A<br>Brachytherapy	No<br>5
Ip(9)<br>2021	M/70/L	Large-cell neuroendocrine carcinoma transformation of prostate cancer<br>Known<br>16	Brain, lung, liver	Elevated retinal mass<br>20/40, 20/400<br>Yes, SRD / No	Clinical (Biopsy from liver metastasis)	Chemotherapy/Immunotherapy<br>Intravitreal injection of chemotherapy/anti-VEGF	Yes<br>9
Özcan(10)<br>2021	M/63/R	Lung adenocarcinoma<br>Known<br>5	Bone	Elevated retinal mass<br>20/32, 20/60<br>No / No	Clinical	Chemotherapy<br>N/A	Yes<br>6
Ucgul(11)<br>2021	M/56/R	Small cell lung carcinoma<br>Known<br>8	Adrenal gland	Elevated retinal mass<br>CF, HM<br>Yes, RRD / No	Clinical	Chemotherapy<br>Radiotherapy	No<br>12
Tetsumoto(12)<br>2020	M/66/R	Laryngeal squamous cell carcinoma<br>Known<br>N/A	Peritoneum<br>(cancerous ascites)	Elevated retinal mass<br>20/400, 20/500<br>No / No	PPV with retinal biopsy	N/A	Yes<br>3
Jorge(13)<br>2020	F/63/L	Lung mucinous adenocarcinoma<br>Unknown<br>0	Liver, lymph node	Elevated retinal mass<br>20/20, 20/20<br>No / No	Clinical (Biopsy from liver metastasis)	Chemotherapy<br>N/A	No<br>8
Yalcinbayir(14)<br>2019	M/54/L	Lung squamous cell carcinoma<br>Known<br>18	Brain, lymph node	Patchy retinal infiltrate<br>CF, N/A<br>Yes, SRD / No	Clinical	Chemotherapy<br>No	No<br>9

Ramtohul(15)<br>2019	M/51/R	Colon adenocarcinoma<br>Known<br>N/A	N/A	Elevated retinal mass<br>CF, N/A<br>Yes, SRD / Yes	PPV with vitreous biopsy	N/A	No<br>6
Whalen(16)<br>2018	M/55/R	Urothelial carcinoma of the bladder<br>Known<br>48	No	Elevated retinal mass<br>20/20, NLP<br>Yes, SRD / Yes	PPV with vitreous biopsy	N/A<br>Radiotherapy/Enucleation	No<br>23
Mano(17)<br>2018	M/69/R	Esophageal carcinoma<br>Known<br>3	Lung, liver, bone, lymph node	Patchy retinal infiltrate<br>20/200, 20/200<br>Yes, TRD / Yes	PPV with vitreous biopsy	No	Yes<br>3
Praidou(18)<br>2017	M/70/R	Hepatocholangiocarcinoma<br>Known<br>1	Widespread	Elevated retinal mass<br>6/24, 6/7.5<br>Yes, SRD / Yes	PPV with retinal biopsy	Chemotherapy<br>Intravitreal injection of anti-VEGF	Yes<br>12
Correa de Mello(19)<br>2017	F/61/R	Breast cancer<br>Known<br>120	No	Elevated retinal mass<br>20/50, N/A<br>Yes, SRD / No	PPV with vitreous biopsy	Targeted therapy<br>Radiotherapy	No<br>15
Vakharia(20)<br>2016	F/80/R	Non-Small cell lung carcinoma<br>Known<br>24	Brain, lung, bone	Patchy retinal infiltrate<br>20/80, N/A<br>No / Yes	Clinical	N/A<br>Radiotherapy	No<br>N/A
Nookala(21)<br>2016	M/56/L	Cecal adenocarcinoma<br>Known<br>25	Lung, liver	Elevated retinal mass<br>N/A, N/A<br>Yes, SRD / No	Clinical	N/A<br>Radiotherapy	No<br>1
Taubenslag(22)<br>2015	M/75/L	Non-Small cell lung carcinoma favored by pathology, but no primary tumor identified<br>Unknown<br>N/A	No	Patchy retinal infiltrate<br>20/25, CF<br>Yes, SRD (complicated RRD later) / Yes	PPV with retinal biopsy	N/A<br>Radiotherapy	No<br>18
Gubbiotti(23)<br>2015	F/52/R	Breast cancer<br>Known<br>138	Brain, lung, liver, bone, lymph node	Patchy retinal infiltrate<br>N/A, N/A<br>No / No	Clinical	Chemotherapy<br>No	No<br>6
Shields(24)<br>2014	F/64/L	Lung carcinoma<br>Known<br>103	Yes (details N/A)	Elevated retinal mass<br>20/50, N/A<br>No / Yes	Fine needle aspiration retinal biopsy	N/A<br>Brachytherapy	No<br>4
Shields(24)<br>2014	M/56/N/A	Esophageal carcinoma<br>Known<br>26	Yes (details N/A)	N/A<br>N/A, N/A<br>Yes / No	Fine needle aspiration retinal biopsy	No	Yes<br>1

Shields(24)<br>2014	F/58/N/A	Breast cancer<br>Known<br>214	Yes (details N/A)	N/A<br>N/A, N/A<br>Yes / No	Fine needle aspiration retinal biopsy	No	Yes<br>1
Shields(24)<br>2014	F/75/L	Breast cancer<br>Known<br>20	Yes (details N/A)	Elevated retinal mass<br>20/40, N/A<br>Yes / No	Fine needle aspiration retinal biopsy	No	N/A (too sick to return)
Singh(25)<br>2014	M/78/R	Small cell lung carcinoma<br>Known<br>N/A	N/A	Patchy retinal infiltrate<br>N/A, N/A<br>No / Yes	PPV with retinal biopsy	N/A	N/A
Payne(26)<br>2012	M/62/N/A	Lung adenocarcinoma<br>Known<br>N/A	N/A	Patchy retinal infiltrate<br>N/A, N/A<br>No / No	PPV with retinal biopsy	N/A<br>Radiotherapy	No<br>10
Coassin(27)<br>2011	F/54/R	Small cell lung carcinoma<br>Known<br>N/A	Brain	Patchy retinal infiltrate<br>N/A, N/A<br>No / Yes	PPV with vitreous biopsy	N/A	No<br>7
Kim(28)<br>2010	F/64/B	Gastric adenocarcinoma<br>Known<br>24	Lung, liver, peritoneum	Punctate retinal infiltrate<br>20/50, 20/40 (both eyes)<br>No / Yes	Fine needle aspiration vitreous biopsy	N/A<br>Radiotherapy	No<br>N/A
Alegret(29)<br>2009	M/15/R	Nasopharyngeal carcinoma<br>Known<br>12	Lung	Elevated retinal mass<br>20/20, 20/20<br>No / No	Clinical	Chemotherapy<br>Radiotherapy	N/A
Rundle(30)<br>2006	F/55/R	Breast cancer<br>Known<br>108	Lung	Elevated retinal mass<br>6/4, 6/5<br>Yes, SRD / No	Clinical	N/A<br>Photodynamic therapy	No<br>2
Sirimaharaj(31)<br>2006	F/60/L	Breast cancer<br>Known<br>36	Brain, lung, spine, lymph node, skin	Punctate retinal infiltrate<br>6/24, 6/12<br>No / Yes	PPV with vitreous biopsy	Chemotherapy<br>Radiotherapy	Yes<br>8
Apte(32)<br>2005	M/39/L	Cecum adenocarcinoma<br>Known<br>3	Lung, liver	Patchy retinal infiltrate<br>20/20, 7/200<br>Yes, SRD / No	PPV with retinal biopsy	N/A<br>Radiotherapy	No<br>3
Rossi(33)<br>2005	M/41/B	Lung adenocarcinoma<br>Known<br>7	Brain, lung, spine, lymph node	Elevated retinal mass<br>N/A, N/A<br>Yes, SRD / No	Clinical	Chemotherapy/Targeted therapy<br>N/A	Yes<br>4
Saornil(34)<br>2004	M/70/L	Gastric adenocarcinoma<br>Known<br>192	Lymph node	Elevated retinal mass<br>N/A, NLP<br>Yes, SRD / No	Enucleation	N/A	Yes<br>23

Truong(35)<br>2002	F/59/L	Breast cancer<br>Known<br>36	Brain, lung, liver	Elevated retinal mass<br>20/70, N/A<br>Yes, SRD / No	Clinical	N/A<br>Radiotherapy	N/A
Gupta(36)<br>2002	M/46/L	Small cell lung carcinoma<br>Unknown<br>0	Brain, lymph node	Elevated retinal mass<br>6/60, HM<br>No / No	Clinical (Biopsy from skin metastasis)	Chemotherapy<br>Radiotherapy	Yes<br>4
Hutchison(37)<br>2001	F/63/L	Large bowel carcinoma<br>Known<br>N/A	Lung	Elevated retinal mass<br>LP, LP<br>Yes, SRD / No	Clinical	N/A<br>Radiotherapy	N/A<br>3, then too sick to return
Cangiarella(38)<br>1996	F/51/L	Esophageal carcinoma<br>Unknown<br>0	Lymph node	Patchy retinal infiltrate<br>N/A, N/A<br>Yes, RRD / Yes	PPV with vitreous biopsy	Chemotherapy<br>Radiotherapy	No<br>4
Spraul(39)<br>1995	F/74/R	Muir Torre syndrome / concurrent breast and colon adenocarcinoma<br>Known<br>N/A	No	Elevated retinal mass<br>20/100, HM<br>Yes, SRD / No	Enucleation	N/A	No<br>6
Leys(40)<br>1990	M/49/L	Small cell lung carcinoma<br>Unknown<br>0	Brain, lymph node	Patchy retinal infiltrate<br>N/A, N/A<br>Yes, SRD / No	Autopsy	N/A	Yes<br>1
Takagi(41)<br>1989	M/45/L	Lung adenocarcinoma<br>Unknown<br>0	Lymph node, adrenal gland	Elevated retinal mass<br>20/40, HM<br>No / Yes	Enucleation	N/A	Yes<br>3
Piro(42)<br>1982	F/67/B	Breast cancer<br>Known<br>N/A (RM noted after autopsy)	No	N/A<br>20/100 (right) HM (left), N/A<br>No / Yes	Autopsy	No	Yes<br>5 (after vitreous metastasis)
Young(43)<br>1979	M/63/L	Lung adenocarcinoma<br>Known<br>4	Bone, lymph node	Patchy retinal infiltrate<br>CF, CF<br>No / Yes	Fine needle aspiration vitreous biopsy	No<br>Radiotherapy	Yes<br>7
Klein(44)<br>1977	M/52/R	Lung squamous cell carcinoma<br>Unknown<br>0	Brain, lung, liver, lymph node, adrenal gland, spleen, kidney, heart	Patchy retinal infiltrate<br>20/30, N/A<br>Yes, SRD / No	Autopsy	N/A	Yes<br>2
Levy(45)<br>1970	M/49/L	Small cell lung carcinoma<br>Known<br>N/A (RM noted after autopsy)	Brain, liver, bone, lymph node, adrenal gland, pancreas, thyroid	N/A<br>N/A, N/A<br>No / No	Autopsy	N/A	Yes<br>N/A

Koenig(46)<br>1963	M/56/R	Bronchogenic lung carcinoma<br>Unknown<br>0	N/A (further study refused)	Elevated retinal mass<br>CF, NLP<br>Yes, SRD / Yes	Enucleation	N/A	Yes<br>14
Duke(47)<br>1959	F/60/R	Uterine adenocarcinoma<br>Known<br>3	Brain (presumed)	Elevated retinal mass<br>20/100, NLP<br>No / No	Enucleation	N/A	Yes<br>6
Kennedy(48)<br>1958	M/51/R	Rectosigmoid annular carcinoma<br>Known<br>9	N/A (presumed generalized metastasis; autopsy refused)	Elevated retinal mass<br>CF, NLP<br>Yes, SRD / No	Enucleation	N/A	Yes<br>8
Smoleroff(49)<br>1934	M/55/R	Gastric carcinoma (with esophageal extension)<br>Known<br>1	Lung, liver, spine, lymph node, adrenal gland	Elevated retinal mass<br>N/A, N/A<br>Yes, SRD / No	Autopsy	N/A	Yes<br>1

References:

1. Chen CL, Chan WC, Chen YC, Hsu YR. Retinal metastasis from systemic carcinoma: clinical, multimodal imaging, and pathological characteristics from a multicenter case series. Graefes Arch Clin Exp Ophthalmol. 2024.
2. Fairbanks AM, Salomao DR, Barkmeier AJ. Metastatic papillary renal cell carcinoma to the retina and vitreous. Am J Ophthalmol Case Rep. 2024;34:102035.
3. Rahman EZ, Shah P, Shah R. Metastatic squamous cell carcinoma masquerading as acute retinal necrosis. Am J Ophthalmol Case Rep. 2023;32:101934.
4. Alabi RO, Haq Z, Bloomer MM, Afshar AR. Clinicopathologic analysis of a case of small cell lung carcinoma metastatic to the retina. Am J Ophthalmol Case Rep. 2023;31:101863.
5. Ustaoglu M, Eagle RJ, Jr., Bloom SM, Wang W, Barr CC, Adeniran JF. Unilateral, Sectoral Retinal Metastasis of Small-Cell Lung Cancer Mimicking Viral Retinitis. Retin Cases Brief Rep. 2023.
6. Marks S, Woods B, Higgins M, Connell P. Small cell lung cancer recurring in the retina. Oxf Med Case Reports. 2022;2022(4):omac038.
7. Gascon P, Matet A, Gualino V, Denis D, Nguyen AM, Papegaey M, et al. CLINICAL FEATURES OF RETINAL METASTASES: New Cases Integrated in a Systematic Review of the Literature. Retina. 2022;42(7):1370-83.
8. Storey PP WBJ, Shields CL. Isolated retinal metastasis from non-small cell lung cancer. Indian J Ophthalmol2021. p. 463.
9. Ip CS, Raizen Y, Goldfarb D, Kegley E, Munoz J, Schefler AC. Peripapillary Neuroendocrine Carcinoma Metastasis: A Novel Approach to Treatment. Ocul Oncol Pathol. 2021;7(5):316-20.
10. Ozcan G, Gunduz AK, Mirzayev I, Saglik A. Presumed Retinal Metastasis from Lung Adenocarcinoma: A Case Report and Literature Review. Case Rep Ophthalmol Med. 2021;2021:6615284.
11. Ucgul AY, Ozdek S, Hasanreisoglu M, Aydin K, Atalay HT. Rhegmatogenous retinal detachment secondary to isolated retinal metastasis from small cell lung carcinoma: An uncommon complication. Eur J Ophthalmol. 2021;31(3):NP11-NP4.
12. Tetsumoto A, Imai H, Miki A, Nakamura M. A Case of Retinal Metastasis of Laryngeal Squamous Cell Carcinoma with Histopathologic Confirmation by Vitrectomy-Assisted En Block Extraction. Retin Cases Brief Rep. 2020;14(2):127-30.
13. Jorge DM, Labarrere M, Rodrigues MW, Shields CL, Jorge R. Simultaneous Choroidal and Retinal Metastases from Lung Carcinoma. Retin Cases Brief Rep. 2020;14(1):90-5.
14. Yalcinbayir O, Gelisken O, Sen F, Yasar S, Alizadegan F. Retinal Metastasis from Squamous Cell Carcinoma of the Lung: A Case Presentation. Retin Cases Brief Rep. 2019;13(2):141-4.
15. Ramtohul P, Denis D, Comet A. Natural Course of a Retinal Metastasis from Colon Adenocarcinoma. Ophthalmology. 2019;126(6):840.

16. Whalen KE, Eagle RC, Jr., Vrabec TR. Case of Metastatic Urothelial Carcinoma of the Retina and Vitreous. Retin Cases Brief Rep. 2018;12(3):177-80.
17. Mano F, LoBue SA, Chang KC, Mano T. Multimodal imaging of retinal metastasis masquerading as an acute retinal necrosis. Int J Retina Vitreous. 2018;4:43.
18. Praidou A, Jacob S, Irion L, Sivaraj R, Groenewald C, Coupland SE, Heimann H. Retinal and vitreous metastases from hepatocholangiocarcinoma. BMC Cancer. 2017;17(1):430.
19. Correa de Mello P, Brasil OFM. Isolated Retinal Metastasis From Breast Cancer. Retina. 2017;37(11):e125-e7.
20. Vakharia P, Yonekawa Y, Randhawa S. Management of Retinal Whitening With Vitritis. JAMA Ophthalmol. 2016;134(7):835-6.
21. Nookala R, Batchu VV, Lee HM, Loghmani A, Chhabra GS. Difficult Diagnosis of Colon Adenocarcinoma Metastasis to Retina: A Case Report and Literature Review. Int J Hematol Oncol Stem Cell Res. 2016;10(3):186-90.
22. Taubenslag KJ, Kim SJ, Attia A, Abel TW, Nickols HH, Ancell KK, Daniels AB. Retinal metastasis from unknown primary: diagnosis, management, and clinicopathologic correlation. Digit J Ophthalmol. 2015;21(4):1-10.
23. Gubbiotti M, Pistilli B, Tudini M, Benedetti G, Galizia E, Rusiello M, Latini L. Retinal metastasis regression with eribulin in a heavily pretreated breast cancer patient. Future Oncol. 2015;11(15 Suppl):17-22.
24. Shields CL, McMahon JF, Atalay HT, Hasanreisoglu M, Shields JA. Retinal metastasis from systemic cancer in 8 cases. JAMA Ophthalmol. 2014;132(11):1303-8.
25. Singh RP, Steinle NC, Bedi R, Kaiser P, Lowder CY. Retinal infiltrates secondary to metastatic squamous cell carcinoma masquerading as infectious retinitis. Retin Cases Brief Rep. 2014;8(4):333-5.
26. Payne JF, Rahman HT, Grossniklaus HE, Bergstrom CS. Retinal metastasis simulating cytomegalovirus retinitis. Ophthalmic Surg Lasers Imaging. 2012;43 Online:e90-3.
27. Coassin M, Ebrahimi KB, O'Brien JM, Stewart JM. Optical coherence tomography for retinal metastasis with unknown primary tumor. Ophthalmic Surg Lasers Imaging. 2011;42 Online:e110-3.
28. Kim CY, Ha CW, Lee SC. Vitreous and retinal metastasis from gastric cancer. Eur J Ophthalmol. 2010;20(3):615-7.
29. Alegret A, Cebulla CM, Dubovy SR, Mutapcic L, Hess DJ, Murray TG. Pediatric nasopharyngeal carcinoma with retinal metastasis. Retin Cases Brief Rep. 2009;3(1):8-11.
30. Rundle P, Rennie I. Photodynamic therapy for solitary retinal metastasis from breast carcinoma. Eye (Lond). 2006;20(12):1410-2.
31. Sirimaharaj M, Hunyor AP, Chan WC, Arnold J. Unsual ocular metastasis from breast cancer. Clin Exp Ophthalmol. 2006;34(1):74-6.
32. Apte RS, Dibernardo C, Pearlman JR, Patel S, Schachat AP, Green WR, Gehlbach P. Retinal metastasis presenting as a retinal hemorrhage in a patient with adenocarcinoma of the cecum. Arch Ophthalmol. 2005;123(6):850-3.
33. Rossi A, Manto A, Maione P, Gridelli C. Synchronous bilateral retinal metastases from lung adenocarcinoma. Tumori. 2005;91(3):287-9.

34. Saornil MA, Blanco G, Sarasa JL, Gonzalez-Sansegundo C, Rabano G. Isolated metastasis of gastric adenocarcinoma to the retina: first presentation of systemic disease. Acta Ophthalmol Scand. 2004;82(1):86-8.
35. Truong SN, Fern CM, Costa DL, Spaide RF. Metastatic breast carcinoma to the retina: optical coherence tomography findings. Retina. 2002;22(6):813-5.
36. Gupta M, Puri P, Jacques R, Rennie IG. Retinochoroidal mass: a presenting feature of metastatic oat cell carcinoma of lung. Eur J Ophthalmol. 2002;12(6):550-2.
37. Hutchison BM, McAllister IL, Barry CJ. Bowel carcinoma metastatic to the retina. Clin Exp Ophthalmol. 2001;29(6):438-9.
38. Cangiarella JF, Suhrland MJ, Cajigas A, Chess J, Koss LG, Berkowitz D, Schlesinger K. Esophageal carcinoma metastatic to the retina. Diagnosis of a case by cytologic examination of intraocular vitreous washings. Acta Cytol. 1996;40(5):995-8.
39. Spraul CW, Lang GE, Grossniklaus HE, Lang GK. Metastatic adenocarcinoma to the retina in a patient with Muir-Torre syndrome. Am J Ophthalmol. 1995;120(2):248-50.
40. Leys AM, Van Eyck LM, Nuttin BJ, Pauwels PA, Delabie JM, Libert JA. Metastatic carcinoma to the retina. Clinicopathologic findings in two cases. Arch Ophthalmol. 1990;108(10):1448-52.
41. Takagi T, Yamaguchi T, Mizoguchi T, Amemiya T. A case of metastatic optic nerve head and retinal carcinoma with vitreous seeds. Ophthalmologica. 1989;199(2-3):123-6.
42. Piro P, Pappas HR, Erozan YS, Michels RG, Sherman SH, Green WR. Diagnostic vitrectomy in metastatic breast carcinoma in the vitreous. Retina. 1982;2(3):182-8.
43. Young SE, Cruciger M, Lukeman J. Metastatic carcinoma to the retina: case report. Ophthalmology. 1979;86(7):1350-4.
44. Klein R, Nicholson DH, Luxenberg MN. Retinal metastasis from squamous cell carcinoma of the lung. Am J Ophthalmol. 1977;83(3):358-61.
45. Levy RM, De Venecia G. Trypsin digest study of retinal metastasis and tumor cell emboli. Am J Ophthalmol. 1970;70(5):778-82.
46. Koenig RP, Johnson DL, Monahan RH. Bronchogenic Carcinoma with Metastases to the Retina. Am J Ophthalmol. 1963;56:827-9.
47. Duke JR, Walsh FB. Metastatic carcinoma to the retina. Am J Ophthalmol. 1959;47(1 Part 1):44-8.
48. Kennedy RJ, Rummel WD, Mc CJ, Hazard JB. Metastatic carcinoma of the retina; report of a case and the pathologic findings. AMA Arch Ophthalmol. 1958;60(1):12-8.
49. Smoleroff JW AS. Metastatic carcinoma of the retina: Report of a case, with pathologic observation. Arch Ophthalmol. 1934;12(3):359-65.
